# Addressing gender and social inequities: the challenge of metabolic control in women with type 2 diabetes in Quito

**DOI:** 10.1080/16549716.2025.2574100

**Published:** 2025-11-05

**Authors:** Karla Margarita Flores-Sacoto, Galo Antonio Sanchez-del-Hierro, Felipe Gonzalo Moreno-Piedrahita Hernández

**Affiliations:** a Facultad de Salud y Bienestar, Pontificia Universidad Católica del Ecuador, Quito, Ecuador; b CERPOP - UMR 1295, Equipe BIOETHICS, Université Toulouse III - Paul Sabatier, Toulouse, France

**Keywords:** Type 2 diabetes, glycemic control, cardiometabolic risk, gender, intersectionality, Ecuador

## Abstract

**Background:**

Type 2 diabetes mellitus (T2DM) is increasingly prevalent in low- and middle-income countries. Metabolic control reflects not only clinical care and individual behaviors but also intersecting social determinants such as sex, education, and insurance status.

**Objective:**

To identify sociodemographic factors associated with poor metabolic control among patients with T2DM at a public outpatient clinic in Quito, Ecuador.

**Methods:**

We conducted a cross-sectional study of adults with T2DM (ICD-10 E10–E14) attending the clinic in the first semester of 2018. Clinical variables included body mass index (BMI), blood pressure, and laboratory results (HbA1c, total cholesterol, HDL-C, LDL-C, triglycerides). Sociodemographic variables were age, sex, education, marital status, and insurance type. Associations with uncontrolled metabolic indicators were examined using bivariate tests and multivariable logistic regression.

**Results:**

A total of 644 patients were included, 53.6% women. Women had significantly higher odds of poor HDL-C control (aOR 1.73; 95% CI 1.26–2.38) and overweight/obesity (aOR 1.96; 95% CI 1.40–2.74) compared with men. No significant sex differences were observed for HbA1c, blood pressure, or LDL-C. Lower education and younger age were also associated with poorer metabolic outcomes.

**Conclusions:**

Metabolic control in this population is shaped by intersecting sociodemographic determinants. These findings highlight the relevance of sex, age, education, and insurance in shaping diabetes outcomes and underscore the value of an intersectional approach for understanding inequities. Equity-oriented strategies that incorporate intersectionality may improve diabetes care in Ecuador. Given the cross-sectional design, findings indicate associations rather than causality.

## Paper context

- **Main findings**: Metabolic control among patients with type 2 diabetes mellitus (T2DM) in an urban Ecuadorian ambulatory clinic is significantly associated with sociodemographic factors, particularly sex, age, educational attainment, and type of health insurance; women of working age with low educational levels and enrolled in general public insurance exhibited the highest rates of poor glycemic and cardiovascular risk control.
- **Added knowledge**: This is the first study in Ecuador to apply an intersectionality-informed framework to assess disparities in metabolic control among patients with T2DM. Reveals how combining structural determinants (insurance type) and individual characteristics (sex, age, education) offers a more nuanced understanding of chronic disease management; it advances empirical evidence from Latin America in applying intersectionality to non-communicable disease outcomes, a gap rarely addressed in regional literature.
- **Global health impact for policy and action**: This is the first study in Ecuador to apply an intersectionality-informed framework to assess disparities in metabolic control among patients with T2DM. Reveals how combining structural determinants (insurance type) and individual characteristics (sex, age, education) offers a more nuanced understanding of chronic disease management; it advances empirical evidence from Latin America in applying intersectionality to non-communicable disease outcomes, a gap rarely addressed in regional literature.

## Background

Type 2 diabetes (T2DM) is one of the most prevalent non-communicable diseases worldwide and a leading cause of morbidity, premature mortality, and disability [1]. It is estimated that 8.5% of adults worldwide have T2DM, though the disease is more prevalent in low- and middle-income countries due to demographic shifts, lifestyle changes, and systemic inequalities. In Ecuador, diabetes is among the top five leading causes of death, and its impact continues to grow alongside structural gaps in the healthcare system.

Although clinical guidelines emphasize the importance of glycemic, lipid, and blood pressure control, achieving adequate metabolic outcomes requires more than individual behavioral changes. The ability to adhere to dietary recommendations, engage in physical activity, and access timely medical care is deeply embedded in broader social, cultural, and economic contexts. Social determinants of health, such as age, sex, education, employment status, and type of health insurance, are known to influence access to care and health outcomes in chronic diseases.

Intersectionality, a concept rooted in feminist and critical race theory, offers a valuable analytical framework for understanding how multiple dimensions of identity and social position interact to produce health inequities [2,3]. Rather than considering determinants in isolation, intersectionality examines how combinations of factors, such as sex and socioeconomic status, jointly affect the lived experience of illness and the structural barriers to disease management [4,5]. This approach has gained increasing attention in public health as a means of addressing layered and persistent health disparities [6].

In the context of diabetes care, intersectional analyses can reveal how structural disadvantages lead to unequal access to treatment, different health-seeking behaviors, and varied clinical outcomes. For example, women with lower levels of education may encounter additional barriers to achieving metabolic control due to limited health literacy, reduced autonomy, and caregiving responsibilities. However, studies using this framework in Latin America; and specifically in Ecuador specifically, are scarce.

This study aims to address this gap by examining the relationship between metabolic control and sociodemographic characteristics among patients with type 2 diabetes mellitus (T2DM) at an ambulatory clinic in Quito, Ecuador. Specifically, we will explore how factors such as sex, age, marital status, education level, and type of insurance relate to clinical outcomes. Using an intersectional approach, this study aims to provide a more nuanced understanding of health disparities in diabetes care and inform equity-oriented public health interventions.

## Methods

### Study design and setting

This was a cross-sectional observational study used data from patients diagnosed with type 2 diabetes (T2DM) who received care at the ‘Eloy Alfaro’ Ambulatory Clinic in Quito, Ecuador. Data were extracted from medical records from to the first semester of 2018. The study was approved by the Research Ethics Committee of the ‘Universidad San Francisco de Quito’ (approval code: 2019-037PG). Written informed consent for participation in the study was obtained.

### Study population and inclusion criteria

The study included adult patients (aged ≥18 years) with a documented T2DM diagnosis, based on ICD-10 codes E10-E14. Patients had to have complete medical records and who consented to the use of their clinical data for research purposes. Patients with type 1 diabetes or other specific types of diabetes were excluded.

The sample size of *N* = 644 is justified by an exhaustive census of all eligible patients with T2DM seen at our secondary care referral facility in South Quito during the study period, which effectively eliminates sampling error. This approach ensures superior internal validity for this specific patient segment. Since the hospital primarily serves mid-to-low-income residents who are covered by the social security system, the sample is highly representative of a vulnerable and understudied group in the capital. Furthermore, the sample size is statistically robust, guaranteeing a margin of error below ±4% for key prevalence estimates. This margin of error is more than adequate for precisely detecting the intersectional inequities affecting metabolic control. Data were obtained from electronic medical records. A total of 31 cases (4.6%) were excluded due to missing key clinical or sociodemographic information. The proportion of missing data was low, thus minimizing the risk of bias. We conducted sensitivity checks to ensure that the excluded cases did not differ substantially from the included cases in terms of age and sex distribution.

### Variables and data collection

The primary outcome was metabolic control, which was assessed using a set of clinical and biochemical parameters based on the 2019 American Diabetes Association standards [1,7]. These included:

- Glycated hemoglobin (HbA1c): uncontrolled if ≥7.5%
- Body mass index (BMI): uncontrolled if ≥25 kg/m^2^
- Systolic blood pressure (SBP): Uncontrolled if ≥130 mmHg for adults under 65 years old or ≥140 mmHg for adults 65 years and older
- Diastolic blood pressure (DBP): Uncontrolled if ≥80 mmHg for adults under 65 years old or ≥90 mmHg for adults 65 years and older.
- Total cholesterol: ≥170 mg/dL
- LDL cholesterol: ≥70 mg/dL
- HDL cholesterol: <40 mg/dL for men and <50 mg/dL for women
- Triglycerides: ≥150 mg/dL

The independent variables included sociodemographic characteristics:

- Age (categorized as 20–64, 65–79, and ≥80 years)
- Sex (female or male)
- Educational level (none, basic, primary, secondary/high school, or university)
- Marital status (single, married, divorced, widowed, or in a common-law union)
- Type of health insurance (public general, voluntary, retiree, dependent, or widowed. Recategorized as active insurance and assigned to individuals who are currently employed or contributing to the social security system through formal work, either as employees or independent contributors (public general and voluntary). In contrast, ‘passive’ insurance refers to individuals who no longer contribute directly, such as retirees, usually over 65 years old, pensioners, and dependents of insured members (e.g. children and spouses), who still retain coverage through their connection to the contributor (retiree, dependent or widowed).

### Statistical analysis

The data were analyzed using IBM SPSS Statistics version 22. Descriptive statistics were used to summarize the population characteristics. Continuous variables were tested for normality using the Kolmogorov – Smirnov test and presented as means with standard deviations or medians with ranges, as appropriate.

Bivariate analyses were conducted to evaluate the association between metabolic control indicators and sociodemographic variables. The Kruskal – Wallis test was used to compare continuous variables across multiple groups, followed by post hoc pairwise comparisons with the Games – Howell test. The Mann–Whitney U test was used to compare differences between two groups. Correlation analyses were performed using Spearman’s rho. A p-value of less than 0.05 was considered statistically significant.

Intersectionality was operationalized by examining sociodemographic subgroups defined by age, sex, education, and insurance type. Stratified analyses and additive logistic regression models were used to identify disparities across groups. Although interaction terms between these sociodemographic factors were tested, they did not improve the models’ fit and were not included in the final models. Thus, this approach therefore emphasizes additive associations rather than compounded intersectional effects.

### Study limitations and potential biases

The cross-sectional design of this study prevents us from making causal inferences about the relationship between metabolic control and the associated sociodemographic factors. Although statistically significant associations were identified, temporality and directionality cannot be determined, so the results should be interpreted as correlational rather than causal. First, this study was conducted at a secondary care facility in urban Quito and primarily served a mid-to-low-income population within the social security system. While this provided a robust census of that specific demographic, the findings may not be directly generalizable to rural Ecuadorian populations due to differing barriers to healthcare, dietary patterns, and social structures. Similarly, the results may not capture the experiences of higher-income urban populations who use private healthcare or those served by the Ministry of Public Health. Consequently, the observed intersectional inequities are specific to this urban context and type of healthcare access. This highlights the need for localized studies in diverse settings.

Second, excluding detailed behavioral variables, such as treatment adherence, dietary patterns, and physical activity levels, had direct implications. While the study successfully identified significant intersectional disparities in metabolic control, it could not elucidate the precise behavioral pathways through which these inequities manifest. This limited the study’s ability to provide a complete mechanistic understanding. This also meant that recommendations for targeted interventions were less specific. Furthermore, the absence of behavioral data suggests the possibility of residual confounding factors, meaning that unmeasured behavioral factors linked to intersectional identities could partly explain the observed differences in metabolic control. These limitations collectively shape the results of the study, indicating the need for future research that incorporates broader geographical representation and a richer array of behavioral variables to achieve a more comprehensive understanding and develop targeted interventions.

Due to the study’s reliance on data from a single public health institution in Quito, Ecuador, selection bias may be present. Patients attending this facility may not be representative of the national population with type 2 diabetes, especially those with private insurance or limited access to healthcare. However, the selected institution serves as a reference for the urban population and provides valuable insight into patients in the public health system.

Information bias may have occurred when recording clinical and sociodemographic data. Although clinical indicators were extracted from medical records, variables such as marital status and education level may be subject to reporting inaccuracies or classification errors.

The analysis was limited by the availability of variables. Potentially important confounders, such as medication adherence, diet, physical activity, and mental health status, were excluded. Omitting these variables may affect the strength and interpretation of the observed associations.

Although we employed an intersectional framework, we constrained the operationalization to stratified and additive logistic regression models. Interaction terms between sociodemographic factors (e.g. sex, age, education, and insurance type) were tested but excluded because they did not improve model fit. This approach represents a partial rather than a full application of intersectionality and should be interpreted as a methodological limitation. Future studies with larger sample sizes and longitudinal designs are needed to fully capture compounded intersectional effects.

## Results

A total of 644 patients with T2DM were included in the analysis. The sample comprised 345 women (53.6%) and 299 men (46.4%), with an average age of 61.3 years (SD ± 11.75). Among them, 269 patients (41.8%) were aged 65 years of age or older, and 412 patients (64%) were married. Regarding insurance coverage, 277 patients (43%) were enrolled in the general public insurance system. Most participants had completed either primary education (38.7%) or secondary education (37.3%).

The median values for the main clinical and biochemical variables are as follows: systolic blood pressure (SBP) 130 mmHg (SD ± 17), diastolic blood pressure (DBP) 75 mmHg (SD ± 11), body mass index (BMI) 30.26 kg/m^2^ (SD ± 5.06), triglycerides 168.5 mg/dL (SD ± 109.9), total cholesterol 190 mg/dL (SD ± 37.8), HDL cholesterol 43 mg/dL (SD ± 12.4), and LDL cholesterol 109 mg/dL (SD ± 32.8). These variables were not normally distributed (Kolmogorov – Smirnov test, *p* < 0.001; see Table 1).

**Table 1.** Key associations between sociodemographic factors and metabolic control.

	Variable	Category	OR (95% CI)*
HbA1c	Insurance type	Active vs Passive	2,65 (1,92–3,66)
	Age group	Adult vs Elderly	4,9 (3,49–6,87)
HDL-c	Sex	Female vs Male	1,73 (1,26–2,38)
LDL-c	Insurance type	Active vs Passive	1,88 (1,08–3,26)
SBP	Age group	Adult vs Elderly	2,06 (1,49–2,86)
	Education	>6 yrs vs ≤6 yrs	0,64 (0,46–0,89)
DBP	Insurance type	Active vs Passive	2,99 (2,05–4,37)
	Age group	Adult vs Elderly	10,3 (6,21–17,2)
	Education	>6 yrs vs ≤6 yrs	0,50 (0,35–0,71)

OR: Odds Ratio; CI: confidence interval; HbA1c: Glycated hemoglobin; HDL-c: High Density Lipoprotein Cholesterol; LDL-c: Low Density Lipoprotein Cholesterol; SBP: systolic blood pressure; DBP: Diastolic blood pressure; * p-value < 0,001.

Note: Full descriptive statistics (medians, SD, percentages) for each biochemical and clinical indicator are provided in Supplementary Table S1.

### Association between age and metabolic indicators

Spearman’s correlation analysis revealed a weak but significant inverse correlation between age and HbA1c levels (rho = −0.184, *p* = 0.001). Conversely, positive correlations were found between age and SBP (*r* = 0.476, *p* < 0.001) and DBP (*r* = 0.367, *p* < 0.001).

The Kruskal – Wallis test showed significant differences in HDL cholesterol (*p* = 0.036), DBP (*p* < 0.001), and BMI (*p* < 0.001) across age groups. Post hoc Games – Howell comparisons indicated the following:

- Higher HDL levels in the 65–79 age group compared to the 20–64 age group.
- Lower DBP in patients aged 65–79 and 80–99 compared to those aged 20–64.
- Lower BMI in older age groups (especially those aged ≥80 years) compared to younger individuals.

### Sex differences

The Mann – Whitney U test revealed that women had significantly higher values of total cholesterol (*p* = 0.028), HDL cholesterol (*p* < 0.001), SBP (*p* = 0.003), and BMI (*p* < 0.001) values than men. Although women had slightly higher HbA1c and triglyceride levels, these differences were not statistically significant.

### Differences by insurance type

Kruskal – Wallis analyses revealed statistically significant differences in SBP (*p* = 0.013), DBP (*p* = 0.019), and BMI (*p* = 0.029) across insurance categories. For instance:

- Patients with general insurance had a lower median SBP than those with retirement- or widowhood-related insurance.
- BMI was significantly higher in the spouse and widow groups than in the retirement insurance group.

### Educational attainment and metabolic control

Significant differences in HbA1c, SBP, and DBP were observed across educational levels (*p* < 0.05). Patients with a university education had the lowest median HbA1c values, while those with a basic education had the highest. Similarly, SBP was significantly lower among individuals with a university education than among those with a high school education. DBP was lower among individuals with a primary education than among those with a secondary education.

### Marital status

Although descriptive differences in metabolic variables were observed by marital status-; higher HbA1c in single individuals and higher BMI in the widowed group- these were not statistically significant according to the Kruskal – Wallis test.

We used multivariable logistic regression models to evaluate the relationship between sociodemographic factors and the probability of poor metabolic control based on seven clinical indicators: systolic and diastolic blood pressure (SBP and DBP), body mass index (BMI), total cholesterol, triglycerides, HDL cholesterol, and LDL cholesterol. All models were adjusted for sex, age, insurance type, educational attainment, and marital status.

**Figure 1.**
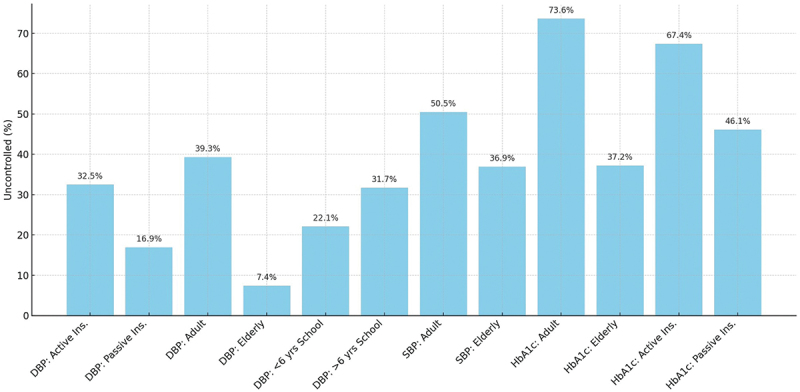
Proportion of uncontrolled metabolic indicators in women with type 2 diabetes by sociodemographic factors.

Analyzing the association between women and sociodemographic factors with poor metabolic control across clinical indicators revealed significant differences in glycemic control, systolic and diastolic blood pressure control among women in different life stages (*p* < 0.001). Figure 1 illustrates the proportion of women with uncontrolled metabolic indicators stratified by age, education, and insurance type. The figure shows that younger women with lower educational attainment and those with active insurance consistently had the highest prevalence of poor control of HbA1c, blood pressure, and lipid parameters. These visual patterns reinforce the regression model results (Table 2), showing that sociodemographic disadvantage is concentrated in specific subgroups of women.

**Table 2.** Multivariable logistic regression models for poor metabolic control.

Outcome (Uncontrolled)	Predictor	aOR* (95% CI)	p-value	Model fit (McFadden R^2^; Hosmer-Lemeshow p)
HbA1c ≥ 7.5%	Age (20–64 vs ≥ 65)	4.9 (3.49–6.87)	<0.001	0.118; 0.42
	Education ( >6 yrs vs ≤6 yrs)	0.64 (0.46–0.89)	0.008	
	Insurance (Active vs Passive)	2.65 (1.92–3.66)	<0.001	
SBP ≥130/140 mmHg	Age (20–64 vs ≥ 65)	2.06 (1.49–2.86)	<0.001	0.103; 0.37
	Education ( >6 yrs vs ≤6 yrs)	0.70 (0.49–0.99)	0.047	
DBP ≥80/90 mmHg	Age (20–64 vs ≥ 65)	10.3 (6.21–17.2)	<0.001	0.141; 0.46
	Insurance (Active vs Passive)	2.99 (2.05–4.37)	<0.001	
LDL ≥70 mg/dL	Insurance (Active vs Passive)	1.88 (1.08–3.26)	0.025	0.076; 0.53
HDL < 40/50 mg/dL	Sex (Female vs Male)	1.73 (1.26–2.38)	<0.001	0.089; 0.49
BMI ≥25 kg/m^2^	Sex (Female vs Male)	1.96 (1.40–2.74)	<0.001	0.097; 0.41

*aOR: adjusted odds ratio; CI: confidence interval. All models adjusted for age, sex, education, marital status, and insurance type.

In the multivariable logistic regression analysis, we found that age group and educational attainment were the strongest predictors of glycemic control in women. Elderly patients had significantly higher odds of achieving HbA1c control than adults did (OR 5.56, 95% CI 3.07–10.05, *p* < 0.001). Similarly, individuals with a university education were more than four times as likely to reach glycemic targets as those with only a basic education (OR 4.66, 95% CI 1.77–12.23, *p* = 0.002). Lower educational levels (primary and secondary) showed positive but non-significant trends. The model explained 12.5% of the variance (McFadden R^2^ = 0.125), indicating that both age and higher education both play critical roles in achieving metabolic control (Table 2). Other sociodemographic factors did not show significant associations.

## Discussion

This study examined the relationship between sociodemographic factors and metabolic control among patients with type 2 diabetes mellitus (T2DM) in an outpatient setting in Quito, Ecuador. The findings reveal that metabolic control is influenced by social determinants as well as biomedical factors. Significant disparities were observed across sex, age, education level, and type of health insurance [8–10]. These results align with previous research indicating that structural and intermediate determinants play a critical role in the management and outcomes of chronic diseases such as T2DM [9].

Notably, an inverse correlation was found between age and HbA1c levels, suggesting that younger patients have poorer glycemic control. This may reflect differences in health-seeking behaviors, adherence, or lifestyle choices, particularly among working-age adults who face competing socioeconomic demands. Conversely, older adults, especially retirees, may benefit from more consistent access to healthcare services, particularly in Ecuador’s healthcare system, where formal retirement is linked to formal insurance coverage [11].

Sex differences were evident in several metabolic indicators. Compared to men, women had presented significantly higher values for BMI, total cholesterol, HDL-c, and SBP. Although elevated HDL-c levels in women may suggest a partial protective profile, their overall metabolic burden remains higher [12–14]. These findings align with literature indicating that women with diabetes often experience worse health outcomes due to compounded social vulnerabilities, including caregiving roles, limited income, and barriers to physical activity and dietary autonomy [15].

Educational level was significantly associated with metabolic control. Patients with higher levels of education tended to have better control of their HbA1c, blood pressure, and lipid levels. Education is widely recognized as a proxy for health literacy, access to information, and self-efficacy- all of which are essential components of self-managing chronic conditions. These findings reinforce the need to strengthen health promotion strategies that are culturally and educationally tailored, especially for populations with limited formal education [16,17].

The type of health insurance also influenced clinical outcomes. Patients with general public insurance tended to have poorer metabolic control than those with retirement or family-based insurance. This reflects inequities within the healthcare system itself, where the type of insurance is associated with differences in the scope and quality of services received. Fragmented systems, long waiting times, and limited availability of multidisciplinary care in public institutions may hinder the continuity of diabetes care. Our findings that poor metabolic control is concentrated among women (Figure 1) underscore the intersection of biological, social, and systemic factors in shaping health outcomes. These findings suggest that structural determinants significantly contribute to inequities in diabetes management.

As shown in Figure 1, the disparities emphasize how intersectional disadvantages converge to shape metabolic outcomes. The clustering of poor control among younger women with active insurance and a low level of education suggests that these groups face structural barriers that extend beyond access to care. While these findings align with our regression models, but the visual representation underscores how inequities manifest simultaneously across multiple indicators, illustrating the layered nature of social disadvantage. Therefore, equity-oriented interventions must address the overlapping determinants of gender, age, education, and insurance.

Although there were no statistically significant associations between marital status and metabolic parameters, descriptive trends suggest that social support structures, such as those provided through marriage or cohabitation, may influence health behaviors and outcomes. Previous studies have emphasized the protective role of social support in managing chronic diseases, a topic that should be further explored in future research.

Importantly, the study emphasizes the usefulness of intersectionality as a conceptual framework for understanding health disparities [18]. Rather than analyzing sociodemographic variables in isolation, intersectionality acknowledges how social identities, such as sex, age, education, and insurance status, interact to shape both exposure to risk and access to care [19,20]. Our findings reinforce this framework by demonstrating that metabolic control among women is not uniformly distributed. Instead, subgroups defined by life stage, insurance affiliation, and educational attainment exhibit markedly different outcomes [21]. Structured around theoretical-methodological debates, social markers, and health policies, the findings demonstrate that intersectionality is a promising and essential analytical resource for understanding and addressing the global challenge of deep social inequalities and disparities in health status [22].

For instance, adult women with less than 6 years of education had higher rates of uncontrolled systolic and diastolic blood pressure. Meanwhile, elderly women with passive insurance were more likely to achieve blood pressure control. The odds ratio for uncontrolled diastolic blood pressure among adult women compared to elderly women was 10.3 (95% CI: 6.21–17.2), highlighting the vulnerability of younger women. Similarly, HbA1c control was significantly poorer among women with active insurance, suggesting barriers not only in access but possibly also in treatment adherence and/or health literacy.

Without an intersectional lens, these patterns would remain invisible, as the aggregated averages might suggest more equitable outcomes. However, our data indicate that specific intersections of sex with other social dimensions are associated with heightened risk clusters, suggesting the need for equity-oriented interventions [23]. This multidimensional perspective is particularly relevant in Latin America, where structural inequalities persist and where health systems are often fragmented [14]. Adopting intersectionality allows public health policies to move beyond universal strategies and address the root causes of disparities in a context-sensitive manner [24].

The finding that younger women with active insurance have higher odds of poor metabolic control requires careful interpretation. This finding requires a broader interpretation beyond healthcare access. Younger women with active insurance are more likely to be employee, and they often face structural inequalities in the workplace, such as precarious employment, lower wages, and limited autonomy in scheduling medical visits [14]. These conditions, combined with caregiving responsibilities within the household, may create additional barriers to treatment adherence, such as difficulty maintaining dietary modifications, physical activity, and consistent follow-up appointments. Therefore, even with formal insurance coverage, these intersecting social and occupational pressures may contribute to poorer metabolic control. This underscores the importance of considering labor market dynamics and gendered caregiving roles when analyzing disparities in diabetes outcomes [23]. It highlights the importance of considering not only healthcare access but also the broader social and occupational context when interpreting disparities in diabetes outcomes.

Our findings align with data from Latin American populations, in which social determinants such as education, sex, and access to health services significantly impact diabetes outcomes [25]. Studies from Mexico and Brazil have shown that lower educational attainment and socioeconomic disadvantage are associated with poorer glycemic control and higher complication rates [26]. Research from Colombia and Chile emphasizes the impact of fragmented insurance systems and sex disparities on disease management and treatment adherence [27].

Regional evidence has demonstrated that health disparities in Latin America are structured through intersecting social determinants. A recent analysis of 14 countries using World Values Survey data confirmed a persistent intersectional gradient of poor self-perceived health. The analysis revealed that the highest risks are concentrated among women of minority or Indigenous ethnicity with low education [28]. Similarly, studies in Mexico have shown that multidimensional poverty and structural stigma disproportionately affect women, especially those engaged in precarious labor conditions or unpaid caregiving roles, which exacerbates barriers to access and adherence [29,30]. Logistic regression analyses in Argentina revealed that gender inequities in self-perceived health were more pronounced among younger adults, individuals with lower education, and those from households with fewer economic resources [31]. Research on older women across the region also underscores the importance of applying intersectionality, revealing how ageism interacts with gender to shape health and social vulnerabilities [32]. Together, this body of evidence situates our results within a broader Latin American epidemiological context, highlighting the cumulative and multidimensional nature of inequities in chronic disease outcomes.

These patterns align with our findings in Ecuador, highlighting that, beyond individual behaviors, broader structural and social inequalities are key drivers of metabolic control in patients with type 2 diabetes across Latin America.

The study highlights the significant impact of sociodemographic factors on metabolic control among patients with type 2 diabetes mellitus in an urban outpatient setting in Ecuador. The findings suggest that sex, age, educational attainment, and type of health insurance are associated with clinical outcomes. These associations may reflect underlying social inequities that extend beyond individual behavior or biological predisposition.

Using an intersectional approach, the analysis reveals how various forms of disadvantage interact to create unique barriers to effective diabetes management. Women, individuals with lower education levels, and those with general public insurance coverage are particularly vulnerable to poor metabolic outcomes. These patterns reflect not only gaps in access to healthcare, as well as broader structural inequities related to gender roles, social support, and resource allocation.

## Conclusion

This study advances our understanding of health disparities in Ecuador by demonstrating significant associations between metabolic control in patients with type 2 diabetes and gender, age, education, and insurance type. The identification of intersectional inequities in metabolic control among women of working age with low educational attainment who are enrolled in general public insurance highlights a critical gap in the equity and effectiveness of diabetes care. Public health strategies must move beyond universal interventions and adopt targeted approaches based on human rights, gender equity, and social justice. This requires prioritizing resources for primary healthcare, implementing health literacy programs, and fostering environments that promote self-care among vulnerable populations.

Furthermore, incorporating the intersectionality framework into health policy design would allow health systems to more accurately identify the complex interactions that perpetuate inequities. In Ecuador, for instance, there is an urgent need to improve comprehensive care for non-communicable diseases in public health networks, guarantee prompt access to medications and laboratory testing, and create community-based programs for prevention and patient support. Incorporating disaggregated data by sex, age, education, and insurance status into health planning processes would facilitate more effective, tailored responses for women with diabetes in urban and rural settings.

The cross-sectional nature of the study warrants a stronger emphasis. Although the study provides valuable insights into associations, it precludes the establishment of causal relationships. To better assess temporal dynamics and the compounded effects of social determinants, future research should incorporate longitudinal cohort designs or repeated measures over time. These approaches would allow for a more robust evaluation of how sociodemographic and structural factors intersect to influence diabetes outcomes.

In order to improve diabetes control in low- and middle-income countries, efforts must move beyond clinical guidelines and embrace equity-focused public health strategies. Policymakers and healthcare providers should incorporate intersectionality into the design and implementation of diabetes prevention and care programs. These programs must be accessible, culturally relevant, and responsive to the complex realities of diverse patient populations.

Future research should explore the causal pathways linking social determinants to clinical outcomes using longitudinal designs and qualitative approaches. In the meantime, addressing the structural roots of inequality is crucial for alleviating the burden of diabetes and establishing fairer, more effective healthcare systems in the region.

## Supplementary Material

C_Supplementary_Table_S1.docx

STROBE.doc

## Data Availability

Data set is available: DOI: 10.6084/m9.figshare.29646644.
