# Supplementary material for: Addressing gender and social inequities: the challenge of metabolic control in women with type 2 diabetes in Quito
Source: Glob Health Action. 2025 Nov 5;18(1):2574100. doi: 10.1080/16549716.2025.2574100 (PMC12590566; doi:10.1080/16549716.2025.2574100)
Supplement: C_Supplementary_Table_S1.docx [file ZGHA_A_2574100_SM9755.docx]

Table S1 Descriptive statistics for biochemical and clinical indicator

|  |  | Uncontrolled | | Controlled | |  |
| --- | --- | --- | --- | --- | --- | --- |
|  |  | Median (SD) | % (n) | Median (SD) | % (n) | OR (CI 95%) |
| HbA1c | Female | 8,3 (1,88) | 57,8 (197) | 6,3 ( 0,67) | 42,2 (144) | 1,12 (0,82-1,53) |
|  | Male | 8,5 ( 2,56) | 55 (164) | 6,3 ( 0,71) | 45 (134) |  |
|  | Active insurante | 8,2 ( 2,36) | 66,9 (240) | 6,1 ( 0,53) | 33,1 (119) | 2,65 (1,92-3,66) |
|  | Pasive insurance | 8,6 ( 1,92) | 43,2 (121) | 6,6 ( 0,7) | 56,8 (159) |  |
|  | Adult | 8,1 ( 2,28) | 72,2 (268) | 6 ( 0,38) | 27,8 (103) | 4,9 (3,49-6,87) |
|  | Elderly | 9 ( 1,92) | 34,7 (93) | 6,69 ( 0,69) | 65,3 (175) |  |
|  | Scholarity < 6 years | 8,5 ( 2,3) | 54,8 (221) | 6,38 (0,68) | 45,2 (182) | 0,83 (0,6-1,15) |
|  | Scholarity > 6 years | 8,3 ( 2,08) | 59,3 (140) | 6,23 ( 0,67) | 40,7 (96) |  |
|  | No formal partner | 8,5 ( 1,87) | 52,6 (112) | 6,4 ( 0,66) | 47,4 (101) | 0,78 (0,56-1,1) |
|  | Formal partner | 8,34 ( 2,37) | 58,5 (249) | 6,2 ( 0,69) | 41,5 (177) |  |
| Total cholesterol | Female | 224 ( 25,3) | 42,4 (146) | 176 ( 21,1) | 57,6 (198) | 1,28 (0,93-1,77) |
|  | Male | 221 ( 27,7) | 36,5 (108) | 172 ( 22,2) | 63,5 (188) |  |
|  | Active insurante | 224 ( 25,6) | 41,2 (149) | 174 ( 19,5) | 58,8 (213) | 1,15 (0,83-1,59) |
|  | Pasive insurance | 221 ( 27,3) | 37,8 (105) | 172 ( 24) | 62,2 (173) |  |
|  | Adult | 224 ( 25,7) | 39,6 (148) | 174 ( 20,5) | 60,4 (226) | 0,98 (0,71-1,36) |
|  | Elderly | 223 ( 27,1) | 39,8 (106) | 172 ( 23,2) | 60,2 (106) |  |
|  | Scholarity < 6 years | 224 ( 27,2) | 38,4 (154) | 173 ( 22,3) | 61,6 (247) | 0,86 (0,62-1,2) |
|  | Scholarity > 6 years | 222 ( 25) | 41,8 (100) | 175 ( 20,6) | 58,2 (139) |  |
|  | No formal partner | 224 ( 24,6) | 36,5 (77) | 171 ( 20,1) | 63,5 (134) | 0,81 (0,58-1,15) |
|  | Formal partner | 223 ( 27,1) | 41,3 (177) | 175 ( 22,5) | 58,7 (252) |  |
| HDL-c | Female | 41 ( 6,78) | 65,1 (220) | 59 ( 10,2) | 34,9 (118) | 1,73 (1,26-2,38) |
|  | Male | 34 ( 5,19) | 51,9 (152) | 47 ( 7,6) | 48,1 (141) |  |
|  | Active insurante | 37,3 ( 7,03) | 58,7 (209) | 53 ( 10,8) | 41,3 (147) | 0,97 (0,71-1,35) |
|  | Pasive insurance | 38 ( 7,12) | 59,3 (163) | 53 ( 10,8) | 40,7 (112) |  |
|  | Adult | 37 ( 6,97) | 61,7 (227) | 52 ( 10,1) | 38,3 (141) | 1,31 (0,95-1,81) |
|  | Elderly | 38 ( 7,22) | 55,1 (145) | 54,8 ( 11,54) | 44,9 (118) |  |
|  | Scholarity < 6 years | 38 ( 7,02) | 58,8 (233) | 53 ( 10,7) | 41,2 (163) | 0,98 (0,71-1,37) |
|  | Scholarity > 6 years | 37 ( 7,11) | 59,1 (139) | 53 ( 11,2) | 40,9 (96) |  |
|  | No formal partner | 39 ( 6,54) | 61,9 (130) | 54,7 ( 10,8) | 38,1 (80) | 1,2 (0,85-1,69) |
|  | Formal partner | 37 ( 7,29) | 57,5 (242) | 52 ( 10,7) | 42,5 (179) |  |
| LDL-c | Female | 114 ( 29,7) | 90,9 (308) | 59 ( 12,8) | 9,1 (31) | 0,96 (0,56-1,67) |
|  | Male | 113 ( 28,9) | 91,1 (267) | 59 ( 11,2) | 8,9 (26) |  |
|  | Active insurante | 113 ( 29,3) | 93,3(332) | 55,5 ( 10,3) | 6,7 (24) | 1,88 (1,08-3,26) |
|  | Pasive insurance | 114 ( 29,3) | 88 (243) | 59 ( 13,2) | 12 (33) |  |
|  | Adult | 113 ( 29,3) | 92,4 (340) | 58 ( 9,98) | 28 (7,6) | 1,5 (0.86-2,58) |
|  | Elderly | 115 ( 29,3) | 89 (235) | 58 ( 13,8) | 11 (29) |  |
|  | Scholarity < 6 years | 114 ( 29,4) | 90,4 (359) | 58,5 ( 13,5) | 9,6 (38) | 0,83 (0,46-1,48) |
|  | Scholarity > 6 years | 113,2 ( 29,1) | 91,9 (216) | 57 ( 8,5) | 8,1 (19) |  |
|  | No formal partner | 107 ( 28,2) | 91,9 (193) | 57 ( 9,63) | 8,1 (17) | 1,19 (0,65-2,15) |
|  | Formal partner | 116 ( 29,7) | 90,5 (382) | 58,5 ( 13) | 9,5 (40) |  |
| Trgyglicerids | Female | 221 (108,8) | 62,2 (214) | 118 (24,8) | 37,8 (130) | 1,11 (0,8-1,52) |
|  | Male | 213 (111,5) | 59,8 (177) | 112 (25,7) | 40,2 (119) |  |
|  | Active insurante | 223 (120,5) | 61,9 (224) | 117 (25,6) | 38,1 (138) | 1,08 (0,78-1,49) |
|  | Pasive insurance | 206 (93,1) | 60,1 (167) | 114 (24,8) | 39,9 (111) |  |
|  | Adult | 219 (117,9) | 63,9 (239) | 119 (25,8) | 36,1 (135) | 1,33 (0,96-1,83) |
|  | Elderly | 211 (95,9) | 57,1 (152) | 111 (24,4) | 42,9 (114) |  |
|  | Scholarity < 6 years | 240 (98,1) | 59,9 (240) | 118 (25,1) | 40,1 (161) | 0,86 (0,62-1,21) |
|  | Scholarity > 6 years | 151 (124,6) | 63,2 (151) | 112 (25,4) | 36,8 (88) |  |
|  | No formal partner | 213 (120,1) | 61,6 (130) | 112 (24,1) | 38,4(81) | 1,03 (0,73-1,45) |
|  | Formal partner | 261 (104,8) | 60,8 (261) | 117 (25,7) | 39,2 (168) |  |
| SBP | Female | 145 (14,7) | 44,6 (154) | 122 (10,1) | 55,4 (191) | 1,31 (0,95-1,79) |
|  | Male | 142 (11,8) | 38,1 (114) | 120 (10) | 61,9 (185) |  |
|  | Active insurante | 140 (11,2) | 42 (153) | 120 (9,51) | 58 (211) | 1,04 (0,75-1,43) |
|  | Pasive insurance | 150 (14,9) | 41,1 (115) | 124 (10,5) | 58,9 (165) |  |
|  | Adult | 140 (11,75) | 48,8 (183) | 120 (8,43) | 51,2 (192) | 2,06 (1,49-2,86) |
|  | Elderly | 151 (14,6) | 31,6 (85) | 125 (10,9) | 68,4 (184) |  |
|  | Scholarity < 6 years | 145 (12,7) | 37,6 (152) | 122 (10,3) | 62,4 (252) | 0,64 (0,46-0,89) |
|  | Scholarity > 6 years | 144 (14,89 | 48,3 (116) | 120 (9,76) | 51,7 (124) |  |
|  | No formal partner | 146 (14,9) | 42,5 (91) | 123 (10,1) | 57,5 (123) | 1,06 (0,75-1,47) |
|  | Formal partner | 143 (12,9) | 41,2 (177) | 120 (10,1) | 58,8 (253) |  |
| DBP | Female | 86 (6,2) | 25,5 (88) | 70 (8,4) | 74,5 (257) | 0,72 (0,51-1,02) |
|  | Male | 85 (7,27) | 32,1 (96) | 72 (8,3) | 67,9 (203) |  |
|  | Active insurante | 86 (6,5) | 37,6 (137) | 70 (7,6) | 62,4 (227) | 2,99 (2,05-4,37) |
|  | Pasive insurance | 90 (7,3) | 16,8 (47) | 71 (8,9) | 83,2 (233) |  |
|  | Adult | 85 (6,3) | 44 (165) | 71 (6,3) | 56 (210) | 10,3 (6,21-17,2) |
|  | Elderly | 91 (6,6) | 7,1 (19) | 70 (9,7) | 92,9 (250) |  |
|  | Scholarity < 6 years | 86 (6,3) | 23,3 (94) | 71 (8,7) | 76,7 (310) | 0,50 (0,35-0,71) |
|  | Scholarity > 6 years | 85 (7,25) | 37,5 (90) | 71 (7,55) | 62,5 (150) |  |
|  | No formal partner | 86 (8,7) | 28 (60) | 72 (8,91) | 72 (154) | 0,96 (0,66-1,38) |
|  | Formal partner | 86 (5,6) | 28,8 (124) | 70 (8,4) | 71,2 (306) |  |
| BMI | Female | 32,4 (5) | 91,3 (312) | 23,2 (1,46) | 8,7 (30) | 1,29 (0,76-2,18) |
|  | Male | 29,7 (3,71) | 89 (266) | 24 (1,19) | 11 (33) |  |
|  | Active insurante | 31 (4,51) | 90,7 (330) | 23,5 (1,32) | 9,3 (34) | 1,13 (0,67-1,91) |
|  | Pasive insurance | 30,4 (4,81) | 89,6 (248) | 23,9 (1,39) | 10,4 (29) |  |
|  | Adult | 31,2 (4,98) | 91,7 (343) | 23,5 (1,37) | 8,3 (31) | 1,5 (0,89-2,53) |
|  | Elderly | 30,1 (3,98) | 88,1 (236) | 23,7 (1,34) | 11,9 (32) |  |
|  | Scholarity < 6 years | 30,9 (4,5) | 89,3 (358) | 23,6 (1,2) | 10,7 (43) | 0,75 (0,43-1,32) |
|  | Scholarity > 6 years | 30,6 (4,86) | 91,7 (220) | 23,5 (1,6) | 8,3 (20) |  |
|  | No formal partner | 31,4 (4,96) | 90,6 (191) | 23,1 (1,7) | 9,4 (20) | 1,07 (0,61-1,86) |
|  | Formal partner | 30,6 (4,45) | 90 (387) | 23,9 (0,99) | 10 (43) |  |
| SD: Standard Deviation, OR: Odds Ratio; CI: confidence interval; HbA1c: Glycated hemoglobin; HDL-c: High Density Lipoprotein Cholesterol; LDL-c: Low Density Lipoprotein Cholesterol; SBP: Systolic blood pressure; DBP: Diastolic blood pressure; BMI: body mass index | | | | | | |
